# Population Genomics Reveals Population Structure and Mating-Type Loci in *Marssonina brunnea*

**DOI:** 10.3390/jof8060579

**Published:** 2022-05-28

**Authors:** Qiang Cheng, Hougang Yang, Junxiang Chen, Lijuan Zhao

**Affiliations:** Co-Innovation Center for Sustainable Forestry in Southern China, Nanjing Forestry University, Nanjing 210037, China; younggang@njfu.edu.cn (H.Y.); 15099439130@163.com (J.C.); zhaolijuan@njfu.edu.cn (L.Z.)

**Keywords:** *Marssonina brunnea*, genome resequencing, population structure, mating-type locus

## Abstract

*Marssonina brunnea* is an important fungal pathogen of poplar trees. We collected 32 *M. brunnea* f.sp. *multigermtubi* (*MbMu*) and three *M. brunnea* f.sp. *monogermtubi* (*MbMo*) isolates from four poplar species in three Chinese regions and performed genome resequencing. An annotation of SNPs of *MbMu* indicated that the SNPs potentially have a functional effect on 69.2% of the predicted genes. Using the SNP dataset of nonredundant isolates, a structure and principal component analysis revealed that *MbMu* and *MbMo* belong to two genetically distinct populations. By contrast, subpopulation structures could not be found among *MbMu* isolates. A neighbor-net analysis and a homoplasy index test provided evidence of recombination among *MbMu* isolates. The short distance (109–174 bp) of linkage disequilibrium half-decay supported the presence of a high level of recombination in the *MbMu* population. The genetic architectures of the *MAT* loci of *MbMu* and *MbMo* were revealed by searching genome assemblies or by homology-based cloning, and a BLAST search verified each isolate carrying one of the two opposite *MAT* loci. This study revealed that the *MbMu* population contains a wide range of functional variants, shows high-frequency recombination, and exhibits heterothallic mating systems, indicating high evolutionary potential and a resultant threat to poplar plantations.

## 1. Introduction

Marssonina leaf spot disease (MLSD) is a widespread and devastating disease of poplar (*Populus* spp.), and its causal agent is *Marssonina brunnea* (Helotiales, Ascomycota) [1]. *M*. *brunnea* was first reported in the USA in 1889 [2], and to date, this fungal pathogen has spread to Europe [3], Asia [4], and Oceania [1], resulting in early defoliation, weakened tree vigor, and reduced biomass accumulation. The isolates of *M*. *brunnea* observed in China are classified into two formae speciales, *M*. *brunnea* f.sp. *multigermtubi* (*MbMu*) and *M*. *brunnea* f.sp. *monogermtubi* (*MbMo*). *MbMu* infects poplar trees of the sections Aigeiros and Tacamahaca, and *MbMo* is hosted by poplar trees of the section Leuce. In Europe, *M*. *brunnea* also has two formae speciales, *M*. *brunnea* f.sp. *trepidae*, which specifically infects *P*. *tremula* of sect. Leuce, and *M*. *brunnea* f.sp. *brunnea*, which infects *P*. *deltoides* and *P*. × *euramerieana* of sect. Aigeiros [5]. According to their specialized host range, the two formae speciales in Europe may represent *MbMo* and *MbMo*, but there is no molecular phylogenetic evidence supporting this association.

Poplars of sect. Aigeros present differences in resistance and susceptibility to *MbMu*. *P*. × *euramericana*, *P*. × *canadensis* and some *P*. *deltoides* are susceptible to *MbMu*; however, some *P*. *deltoides* genotypes have complete resistance. A recent study has shown that two large-effect quantitative trait loci were responsible for the differentiation of *MbMu* resistance in *P*. *deltoides* [6]. *P*. *deltoides* and *P*. × *euramericana* were introduced into China in the 1970s from North America and Europe, and these poplars and their hybrids are widely cultivated in the country. In particular, elite cultivars with *MbMu* resistance have been planted on over 4 million acres in the lower-middle reaches of the Yangtze river basin, where MLSD is prevalent [6].

Widespread poplar planting with resistant cultivars in China poses the risk of resistance to *M*. *brunnea* being overcome. However, to date, there has been limited research on the *M*. *brunnea* population and its means of reproduction. With randomly amplified polymorphic DNA (RAPD) markers, Han et al. analyzed the genetic diversity of 37 *MbMu* and five *MbMo* isolates from multiple geographic regions and hosts. The results supported the genetic classification of the formae speciales, and no significant associations between genetic divergence and geographic regions/hosts were found within the *MbMu* population [4].

The teleomorph (sexual stage) of *M*. *brunnea* is referred to as *Drepanopeziza tremulae* and the apothecia of *D*. *tremulae* has been observed once on naturally infected leaves, but not on an artificial medium [7]. In the Ascomycota, a single mating-type (*MAT*) locus, which has alternative forms (idiomorphs), *MAT1-1* and *MAT1-2*, controls sexual reproduction. The *MAT1-1* idiomorph contains the *MAT1-1-1* gene, encoding an alpha-box protein, and the *MAT1-2* idiomorph carries the *MAT1-2-1* gene, encoding a high mobility group-motif protein. Strains of heterothallic fungi contain one idiomorph and are required to mate with a member containing the opposite idiomorph for sexual production. In contrast, homothallic fungi contain both *MAT1-1-1* and *MAT1-2-1* genes within a single individual, which allows for self-crossing [8]. The *MAT* locus and mating system of *M*. *brunnea* remain unknown to date.

The whole-genome sequence of one *MbMu* isolate, MB_m1, which is a 51.95 Mb genome assembled from 89 scaffolds, was reported in 2012 [9], but no *MbMo* genome has been reported. Here, we sequenced 32 *MbMu* and three *MbMo* isolates and conducted population genomic analyses to explore (i) the population structure of *M*. *brunnea*, including whether an admixture between *MbMu* and *MbMo* exists, and whether there are host or geographic subpopulations in the *MbMu* population; (ii) genetic recombination among *MbMu* isolates; and (iii) the mating system of *M*. *brunnea*.

## 2. Materials and Methods

### 2.1. Isolates Collection

**Figure 1:**
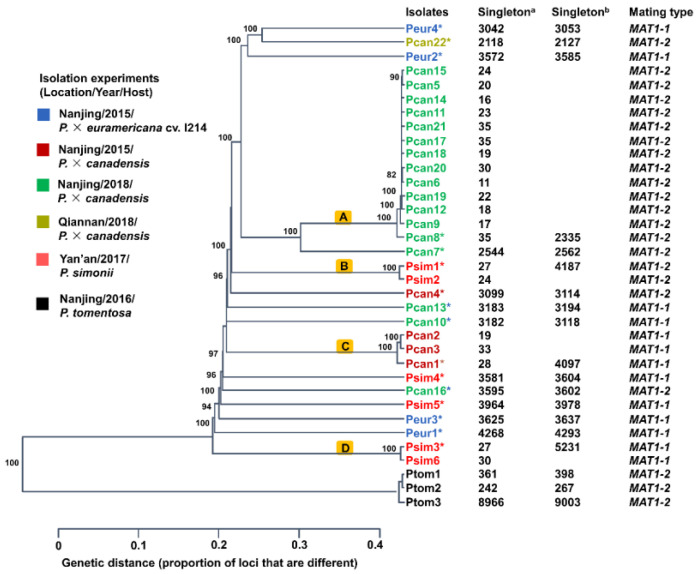
Phylogenetic analysis of 35 *M*. *brunnea* isolates. The UPGMA tree was inferred by Poppr with 1000 bootstrap replicates. Different isolation experiments are labeled in different colors. Pcan, *P*. *canadensis* of sect. Aigeiros; Peur, *P*. × *euramericana* cv. I214 of sect. Aigeiros; Psim, *P*. *simonii* of sect. Tacamahaca; Ptom, *P*. *tomentosa* of sect. Leuce; Nanjing, 118°77′ E, 32°04′ N; Yan’an, 109°28′ E, 36°36′ N; Qiannan, 107°47′ E, 27°07′ N; Singleton ^a^, singletons identified in the SNP dataset of 35 isolates; Singleton ^b^, singletons identified in the SNP dataset of 19 nonredundant isolates; Asterisk, 19 nonredundant isolates; and mating-type, a single *MAT* locus identified in genome assembly of each isolate. Clades (A, B, C, and D), redundant clones.

*MbMu* isolates were collected from infected leaves of *P*. × *euramericana* cv. I214 during 2015 in Nanjing (118°77′ E, 32°04′ N), located in the southeast of China; *P*. × *canadensis* during 2015 and 2018 in Nanjing, *P*. × *canadensis* during 2018 in Qiannan (107°47′ E, 27°07′ N), located in southwest China; and *P*. *simonii* in Yan’an (109°28′ E, 36°36′ N), located in northwest China. *MbMo* isolates were collected from infected leaves of *P*. *tomentosa* located in Nanjing during 2016 (Figure 1 and Table 1). The infected leaves were cut into ~5-mm segments, surface-disinfected in 0.1% mercuric chloride, and washed in sterile distilled water; the segments were placed on potato dextrose agar (PDA) and incubated at 25 °C for 2 weeks. After 20 days, colonies with asexual conidia developed on the edge of the leaf disk. Single-spore isolates were produced by diluting the conidia and spreading them on water agar and later transferring single, germinating conidia to PDA.

### 2.2. DNA Extraction and Genome Sequencing

The mycelia of single-spore isolates grown on cellophane-overlaid PDA plates were collected and ground in liquid nitrogen. DNA was extracted using the DNAsecure Plant Kit (Tiangen, Beijing, China). DNA quality was verified on 1.5% agarose gels, and DNA concentration was measured using a Qubit 3.0 Fluorometer (Invitrogen, Carlsbad, CA, USA). The libraries were made with a NEB Next Ultra DNA Library Prep Kit for Illumina (New England Biolabs, Ipswich, MA, USA) with an insert size of 350 bp following the manufacturer’s protocol. The DNA libraries were sequenced on an Illumina HiSeq 2500 sequencer (Illumina, San Diego, CA, USA) producing 150 bp paired-end reads. The raw reads were cleaned by removing the adapter sequences, low-quality sequences (Phred quality < 5), and any reads with more than 10% unknown sequences.

### 2.3. Read Mapping, SNP Calling, and Summary Statistics

The clean reads were mapped onto the *MbMu* MB_m1 reference genome [9] using a Burrows-Wheeler Aligner (BWA) v0.7.15 [10]. Aligned reads were sorted and duplicates dislodged with Samtools 1.6 [11]. SNP calling was performed using GATKs HaplotypeCaller [12] with the ploidy set to one, according to the Best Practices guidelines. SNPs were filtered using the following parameters: QD < 2.0, MQ < 40.0, FS > 60.0, and DP < 20. Only biallelic SNPs supported by more than five aligned reads and SNPs presenting in all isolates were kept in the final variant call file. F_ST_ values were calculated with DNAsp 6 [13]. SNPs were annotated using SnpEff5.0 [14].

### 2.4. Population Structure

The structure of *M*. *brunnea* was analyzed with fastSTRUCTURE 1.0 software [15], with 10 replicates for each K value (population cluster) from 1 to 10. The optimal K value was chosen using the “chooseK.py” script and visual inspection. The optimal number of clusters was also determined on the lowest cross-validation error of K values from 1 to 10 generated by ADMIXTURE software [16]. A principal component analysis (PCA) was run using the glPCA function of the R package in Poppr v3.2.4 [17]. A minimum spanning network (MSN) using bitwise genetic distance was implemented with the poppr.msn function of Poppr v3.2.4, to show the relationships among all isolates in the population.

### 2.5. Phylogeny and Recombination Analysis

Phylogenic analyses that were estimated using the SNP dataset were conducted with Poppr v3.2.4 using the unweighted pair group method with arithmetic mean (UPGMA) and 1000 bootstrap resamples and with SplitsTree 4 [18] using a neighbor-net algorithm with the uncorrected P distance. A phylogenetic analysis estimated using *MAT1-2* idiomorph sequences was implemented in Mega 7.0 [19] using the maximum-likelihood method with the Tamura–Nei model and 1000 bootstrap replicates.

The presence of recombination was tested with the homoplasy index (PHI)-test implemented in SplitsTree 4. Linkage disequilibrium (LD) decay was calculated for all pairs of SNPs within 300 kb using PopLDdecay [20].

### 2.6. Genome Assembly and MAT Loci

The cleaned reads from each isolate were assembled using SOAPdenovo [21], and the completeness assessment of the assembled genomes was undertaken using BUSCO v3 against the fungi_odb9 dataset [22]. The *MAT1-1* and *MAT1-2* loci of *MbMu* and *MAT1-2* loci of *MbMo* were revealed by tBLASTn searches against these assemblies using the representative *MAT* genes (*MAT1-1-1* and *MAT1-2-1*) of Pezizomycotina as query sequences [8]. The *MAT1-1* locus of *MbMo* was obtained by homology-based cloning. The middle section of *MbMo MAT1-1* was amplified by primers designed according to *MAT1-1-3* and *MAT1-1-1* of *MbMu* (5′-CAGATCAGCGAAGTAGTTGTCG-3′ and 5′-ACTGGCGTAAGGATCATTGAGT-3′, respectively); then, according to the middle section of *MbMo MAT1-1*, endonuclease DNA lyase gene (*APN2*) and cytoskeleton assembly control protein gene (*SLA2*) of *MbMo*, two pairs of primers (5′-GGGACTTTTATTTGGAGCAGTG-3′/5′-GCTGCCGGATCTTTCTCTACTA-3′ and 5′-AGTACCTGTTGAACCACCCAAT-3′/5′-GAAGGTATCGTACCAGGAGGAG-3, respectively) were designed to amplify the flanking regions. We amplified the above-mentioned sections from *MbMo* Ptom5 genomic DNA using LA Taq (Takara, Dalian, China). The polymerase chain reaction (PCR) procedure was as follows: initial denaturation at 94 °C for 3 min, followed by 36 cycles at 94 °C for 30 s, 56 °C for 30 s, 68 °C for 4 min. The PCR products were cloned into pMD-19T vector (Takara, Dalian, China) and sequenced. Full-length *MbMo MAT1-1* locus was assembled from overlapping DNA fragments.

## 3. Results

### 3.1. Resequencing and SNP Calling of MbMu and MbMo

We obtained 2.00–3.26 Gb of clean DNA data from 32 *MbMu* isolates and mapped them to the reference genome (*MbMu* MB_m1). Most MB_m1 genomic regions (88.99% to 97.92%) were covered by at least one read, and the mean depth was 36.9-fold (30.16–47.27 fold) (Table 1). We also sequenced three *MbMo* isolates and generated 16.18, 3.51, and 3.81 Gb of clean data, respectively, which covered 47.38–48.25% of the MB_m1 genomic regions (mean depth 30.88–127.28 fold). Based on the mapping results, 1,139,808 biallelic SNPs without missing data were obtained. Phylogenetic analysis using this SNP dataset revealed that 20 isolates in four clades (A, B, C, and D) showed very short genetic distances, and isolates from each clade were derived from the same isolation experiments (same location, host, and time). Furthermore, the number of SNP singletons of these isolates was low (11–35) (Figure 1), indicating that they were redundant clones.

After removing the redundant isolates, we obtained a SNP dataset of 19 isolates containing 1,140,345 biallelic SNPs without missing data, in which 32,941–43,147 SNPs were identified in 16 *MbMu* isolates and 1,023,223–1,023,392 SNPs were identified in three *MbMo* isolates (Table 1). The number of singletons of each isolate ranged from 267 to 5231 (Figure 1). SNP calling with the 16 nonredundant *MbMu* isolates obtained a dataset containing 430,649 biallelic SNPs without missing data. SNP annotation of the SNP dataset of 16 nonredundant *MbMu* isolates showed that 864 gene structures had been greatly influenced by changes in splicing (481) or resulted in stop codon gain (1116) and loss (104), and 6584 gene coding sequences were changed due to non-synonymous SNPs (42,359). That is, 6938 genes possessing potential functional variations accounted for over 69.2% of the predicted genes (10,027) in *M*. *brunnea* MB_m1.

### 3.2. Population Structure Analysis of MbMu and MbMo

**Figure 2:**
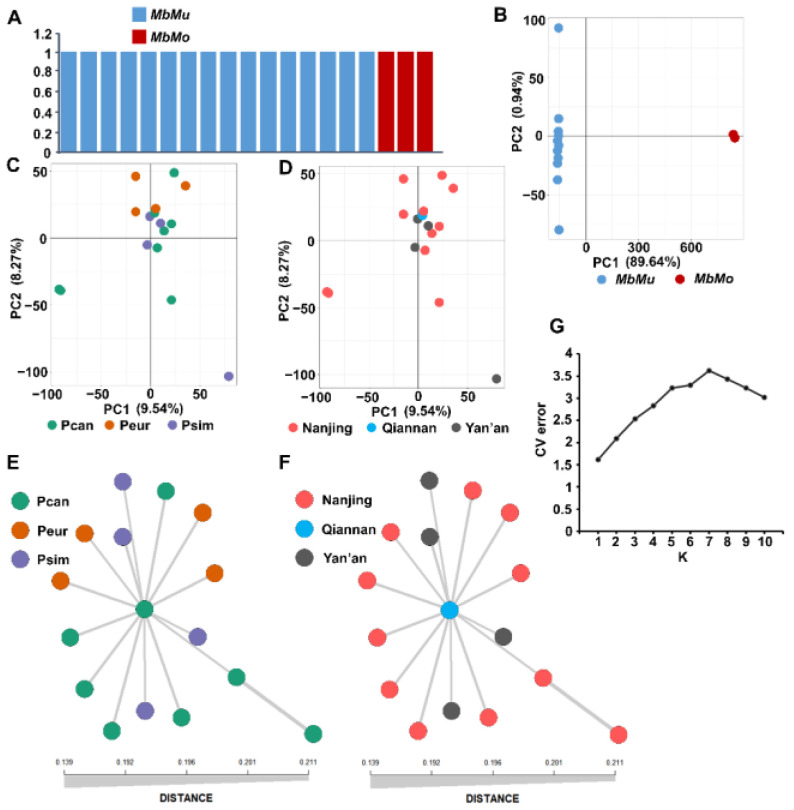
Population structure analysis of *M*. *brunnea* isolates. (**A**) Structure plot inferred by fastSTRUCTURE with the SNP dataset of 19 nonredundant *M*. *brunnea* isolates. K = 2. y-axis quantifies cluster memberships. (**B**) PCA of 19 nonredundant *M*. *brunnea* isolates. (**C**,**D**) PCA of 16 nonredundant *MbMu* isolates. The color of the circle corresponds to the hosts (**C**) and sampling locations (**D**). (**E**,**F**) MSN plots of 16 nonredundant *MbMu* isolates. The color of the circle corresponds to the hosts (**E**) and sampling locations (**F**). (**G**) Cross-validation (CV) error (y-axis) for K between 1 and 10. The estimation was generated by ADMIXTURE software.

The above phylogenetic analysis showed that isolates from *MbMu* and *MbMo* belong to distinct clades. The genetic distance between the two formae speciales is larger than that within each clade. With the SNP dataset of 19 nonredundant isolates, fastSTRUCTURE analyses identified K = 2 as the optimal population number, in which isolates from *MbMu* and *MbMo* were separately grouped, and no admixture signal was found (Figure 2A). In the PCA, one principal component accounted for 89.64% of the total variance in the data, which clearly distinguished isolates from the two formae speciales (Figure 2B). Furthermore, the huge genetic differentiation between *MbMu* and *MbMo* was supported by an F_ST_ value of 0.91.

In contrast with the SNP subset of 16 nonredundant *MbMu* isolates, the PCA showed that the first and second principal components explained only 9.54% and 8.27% of the variance, respectively, and no clustering associated with host or sampling location was observed (Figure 2C,D). The minimum spanning network (MSN) plots also showed that the *MbMu* population was not differentiated according to host or location (Figure 2E,F). fastSTRUCTURE analysis assigned all samples into a single cluster (the optimal population number K = 1), and based on the lowest cross-validation error, admixture software determined that the most likely number of populations was 1 (Figure 2G). Furthermore, the F_ST_ values among different host and geographic *MbMu* groups were 0.0017–0.01. Therefore, all of these results supported no subdivision within the *MbMu* population.

### 3.3. Recombination in the MbMu Population

**Figure 3:**
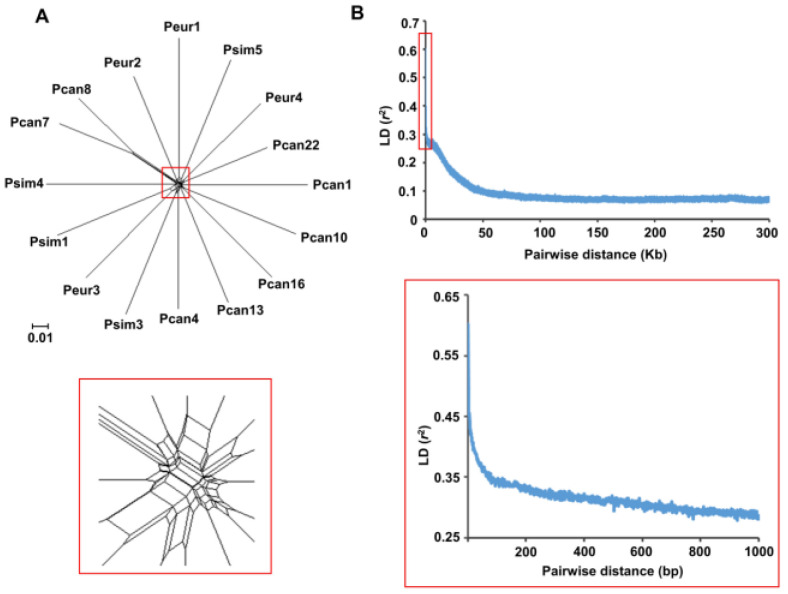
Recombination in the *MbMu* population. (**A**) Neighbor-net phylogenetic network of the 16 nonredundant *MbMu* isolates. Lower panel, local enlargement. (**B**) LD decay in *MbMu*. The squared correlation coefficient (r^2^) between pairs of SNP loci was plotted against the physical distance along the genome. Lower panel, local enlargement.

The lack of a subpopulation structure among the *MbMu* isolates might be explained by frequent recombination. The neighbor-net analysis of *MbMu* isolates showed a multifurcating phylogenetic tree with complicated reticulations in the base, implying the existence of recombination events (Figure 3A). Furthermore, a PHI-test implemented in SplitsTree provided statistically significant evidence for recombination (*p* = 0.0). We then measured the frequency of genetic recombination by LD decay. The LD value decayed to half between the maximum and minimum (LD50) at 109–174 bp, indicating high-frequency recombination in the *MbMu* population (Figure 3B).

### 3.4. MAT Loci of M. brunnea

To identify the *MAT* loci of *M. brunnea*, we assembled the genomes of 35 isolates and obtained 33.8–40.99 Mb assemblies of *MbMu* and 37.06–50.87 Mb assemblies of *MbMo*. The completeness of the assemblies was estimated to be 91%–99%. Using a BLAST search against assemblies with *MAT* genes of Pezizomycotina, a single *MAT1-1-1* gene was identified in 13 *MbMu* isolates, a single *MAT1-2-1* gene in 19 *MbMu* isolates, and a single *MAT1-2-1* gene in each *MbMo* isolates (Figure 1).

**Figure 4:**
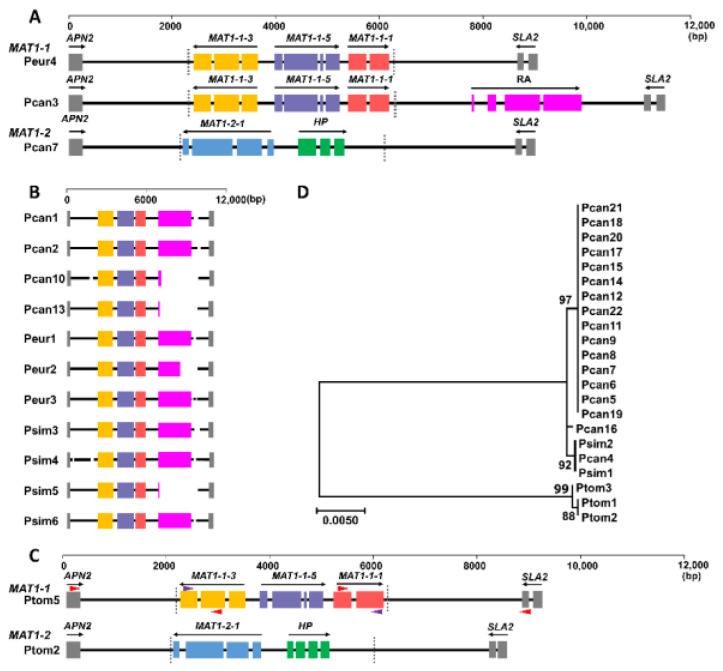
The *MAT* loci of M. brunnea. (**A**) Structures of the *MAT1-1* and *MAT1-2* loci of *MbMu*. Solid boxes represent the coding regions of the predicted genes interrupted by introns. Black arrows indicate the orientations of the coding sequences. Dotted lines mark the sizes of the unique sequences of the idiomorphs. (**B**) A simple diagram of *MAT1-1* loci of 11 *MbMu* isolates. The correspondence of box colors and predicted genes are consistent with that seen in (A). The interruption of the solid line represents the gap in the assembly. (**C**) Structures of the *MAT1-1* and *MAT1-2* loci of *MbMo*. Triangles represent primers used to clone *MAT1-1* of *MbMo*. Purple triangle, primers designed based on *MAT1-1* sequence of *MbMu*; red triangle, primers designed based on *MAT1-1* sequence of *MbMo*. (**D**) Phylogenetic tree constructed with *MAT1-2* idiomorph sequences. The tree was constructed using the maximum likelihood method and tested with 1000 bootstrap replicates.

One complete *MbMu MAT1-1* locus was found in the Pcan3 assembly, of which a retrotransposon structure, i.e., a gene encoding a complete RNaseH domain of reverse transcriptase (RA), was located in the region between *MAT1-1-1* and *SLA2* (Figure 4A). The *RA* was moderately repetitive in the MB_m1 genome, with 17 copies (E-value < 1E-50, identity > 81%). The *MAT1-1* loci of 11 *MbMu* isolates had a gap flanking part of the *RA* sequence (Figure 4B), indicating that these isolates may also carry *RA* in their *MAT1-1* loci. By contrast, the complete *MAT1-1* locus of the Peur4 assembly did not contain any of the *RA* sequence. Three protein-coding genes were predicted in each *MbMu MAT1-1* locus, *MAT1-1-1*, *MAT1-1-3*, and *MAT1-1-5* (Figure 4A,B).

The *MAT1-2* loci of *MbMu* isolates were well assembled, with 12 complete *MAT1-2* loci, and seven *MAT1-2* loci disrupted in short repetitive sequences. Each *MAT1-2* locus included two genes, *MAT1-2-1* and a hypothetical protein gene (HP). HP homologs were found in the *MAT1-2* locus of *M. coronaria* (49.8% amino acid sequence identity) and Rhynchosporium agropyri (30%).

Through homologous cloning and sequencing, a complete *MAT1-1* locus in Ptom5 was revealed; the *MAT1-1* locus of Ptom5 also included *MAT1-1-1*, *MAT1-1-3*, and *MAT1-1-5*, which had a high identity of nucleotide sequences with homologs of *MbMu* (98%, 97%, and 96%, respectively) and no transposon sequence. The *MAT1-2* locus structure of *MbMo* was consistent with that of *MbMu*, with high similarity to *MAT1-2-1* and HP (91% and 82% identity, respectively) (Figure 4C).

The sizes of the *MAT1-1* and *MAT1-2* idiomorphs of *MbMu* were 4204 bp and 3646–3727 bp, and those of *MbMo* were 4210 bp and 3664 bp, respectively. The *MAT1-1* idiomorphs of different *MbMu* isolates had 100% identity, and there were 150 DNA polymorphic sites between the *MAT1-1* idiomorphs of *MbMu* and *MbMo*. Alignment of the idiomorphs of *MAT1-2* revealed nine DNA polymorphic sites within the *MbMu* isolates, two DNA polymorphic sites within the *MbMo* isolates, and 175 DNA polymorphic sites between *MbMu* and *MbMo*. A phylogenetic analysis of the *MAT1-2* idiomorphs revealed that the *MbMu* isolates were divided into two highly supported clades and a single branch, and this phylogenetic relationship was similar to that generated by all SNPs (Figure 4D).

## 4. Discussion

The primary goal of this study was to explore the population structure of *M*. *brunnea* distributed in China using a population genomics approach. In China, there are two *M*. *brunnea* formae speciales, *MbMu* and *MbMo*, which are exclusively derived from Aigeiros/Tacamahaca and Leuce poplar trees, respectively. We found that the sequencing reads of *MbMo* covered 47.38–48.25% of the reference of genome *MbMu*, while two other *Marssonina* fungi, *M*. *coronaria* and *M*. *rosae* that infect apple and rose, respectively, covered less than 2% of the *MbMu* genome (data not shown), indicating a highly similar genomic content between *MbMo* and *MbMu*.

The early analysis of the *M*. *brunnea* population structure with 78 RAPD polymorphic sites supported the suggestion that *MbMu* and *MbMo* are two genetically distinct populations, while this analysis also revealed that an isolate from a Leuce poplar tree admixed polymorphisms of *MbMu* and *MbMo* [4]. The poplar hosts of *MbMu* and *MbMo* are distributed in overlapping regions of China. For example, the distance between poplar trees (*P*. × *euramericana* cv. I214, *P*. × *canadensis*, and *P*. *tomentosa*) involved in the Nanjing sampling experiments was less than 500 m. The trees were more than 40 years old, and MLSD had been recorded on them since the 1990s. Therefore, it is noteworthy to test the admixture between closely related *MbMu* and *MbMo* that have a long-term overlapping distribution. In this study, analyses with more than 1 million SNPs, including phylogenic analysis, structure analysis, and PCA, clearly distinguished isolates of *MbMu* and *MbMo* (Figure 1 and Figure 2A,B), indicating that even if there was individual recombination between *MbMu* and *MbMo*, there was no significant gene flow at the population genome level.

When the SNP dataset containing all isolates was used for phylogenetic analysis, four poorly differentiated clades within the *MbMu* population were observed (Figure 1), and for structure analyses, the isolates from each clade were also assigned to different clusters (data not shown). However, the isolates of each clade always exhibited the same mating type and carried a very small number of SNP singletons (Figure 1). Therefore, these poorly differentiated clades were likely to be separately composed of redundant clones generated by asexual reproduction of one genotype. After removing redundant clones, the phylogenetic relationships of the *MbMu* isolates formed a star-like multifurcating tree without any tight clades (Figure 2E,F and Figure 3A), and PCA and structure analysis supported one genetic cluster of all *MbMu* isolates without the subdivision of host and geographic region (Figure 2C–E). This conclusion was consistent with that of the early *M*. *brunnea* population research conducted by Han et al. [4].

Since chromosomal crossover that breaks linkage leads to an LD decrease with the distance between the two loci, LD decay is often used to analyze the frequency of genetic recombination in population genomic research. According to the LD decay study on multiple fungal populations by Nieuwenhuis et al., the obligately outcrossing mushroom *Schizophyllum commune* has the lowest LD50 at 110 bp, fungi with mixed reproductive modes have LD50 values of 130–27,010 bp, and highly clonal *Candida albicans* has an LD50 of 162,100 bp [23]. This indicated that the *MbMu* population (LD50 = 109–174 bp) has a very high recombination frequency. Therefore, the lack of a subpopulation of *MbMu* may be explained by the high frequency of genetic recombination between isolates. In addition, poplars of sect. Aigeros, introduced in the 1970s, were first bred in a limited area, such as Nanjing and northern Jiangsu province, where MLSD was prevalent. This allowed the genetic admixture between isolates or putative subpopulations. Then, the frequent transportation of nursery stock that bore *MbMu* may have resulted in the current countrywide admixture of *MbMu* isolates.

A BLAST search against assemblies revealed that single isolates only carry one of the two opposite *MAT* idiomorphs, implying a heterothallic system in *M*. *brunnea*. Due to recombination suppression in the *MAT* loci of heterothallic fungi [24], the phylogenetic analysis based on idiomorph sequences should be not affected by admixture of recombination. Using this approach, we found that all the *MAT1-1 MbMu* isolates may come from the same ancestor (100% identity within idiomorph), while the *MAT1-2 MbMu* isolates may be derived from three different ancestors. Whether these inferred ancestors represent *MbMu* isolates before genetic admixture needs further verification using isolates collected from natural forests far from areas to which seedlings may have been transported.

The structures of the *MAT* loci of many Helotiales fungi have been revealed; for example, *Monilinia fructicola*, *M*. *laxa*, *M*. *fructigena* [25], *Hymenoscyphus fraxineus*, *H*. *albidus* [26], *Botrytis cinerea*, *Sclerotinia sclerotiorum* [27], *S*. *minor* [28], *Phialocephala scopiformis* [29], *Rhynchosporium commune*, *R*. *agropyri* [30], and *M*. *coronaria* [31], in which *Rhynchosporium* spp., *M*. *coronaria*, and *M*. *brunnea* have the same set of *MAT* homologous genes. *M*. *brunnea* does not have truncated *MAT1-1-1* (*dMAT1-1-1*) fragments in the *MAT1-2* locus, while this *dMAT1-1-1* is conserved in other heterothallic fungi in the order Helotiales, which was presumed to be a trace of evolution from the homothallic *MAT1* locus to the heterothallic locus [27]. In addition, we found a transposon component, an RNaseH gene (*RA*), in the *MAT1-1* loci of most *MbMu* isolates. *RA* was not included in the idiomorph region, because the sequence between *MAT1-1-1* and *RA* was conserved in the *MAT1-2* locus. However, we also found that there was no *RA* sequence in the *MAT1-2* loci of all *MbMu* isolates, reflecting the expansion of the range of recombination suppression beyond the *MAT* idiomorph region in *MbMu*.

## 5. Conclusions

In conclusion, we confirmed that *MbMu* and *MbMo* are genetically distinct populations at the population genome level. We found a wide range of functional variants, high-frequency recombination, and heterothallic mating systems in *MbMu*, implying that *MbMu* is able to efficiently create new genotypes through outcrossing. Considering that there are extensive plantations of Aigeiros poplars in China, which consist of a small number of *MbMu* resistant clones, the evolution of the *MbMu* population to overcome resistance is likely to be an important risk.

## Figures and Tables

**Table 1:** *M*. *brunnea* isolates sampled in this study and associated data.

Isolate ^a^	Sampling Experiment ^b^	Clean Data (Gb)	Assembly (Mb)	Genome Coverage	Average Read Depth	SNP ^c^	SNP ^d^	SNP ^e^
Peur1 *	1	2.36	35.52	95.51%	32.97	42,314	40,633	99,752
Peur2 *	1	2.66	36.06	95.64%	38.14	39,390	38,066	88,244
Peur3 *	1	3.12	35.79	96.82%	44.05	42,252	40,766	93,741
Peur4 *	1	2.28	36.01	96.37%	33.48	39,157	37,698	88,639
Pcan1 *	2	2.04	33.8	92.65%	32.61	42,501	40,961	94,116
Pcan2	2	2.79	36.18	96.58%	39.06	42,438		
Pcan3	2	2.34	35.06	93.59%	36.15	42,455		
Pcan4 *	2	3.26	35.74	93.69%	47.27	40,182	38,829	91,156
Pcan5	3	2.56	36.86	93.83%	40.62	38,552		
Pcan6	3	2.72	35.22	92.97%	41.42	38,531		
Pcan7 *	3	2.03	35.34	96.24%	31	39,548	38,055	94,022
Pcan8 *	3	2.15	34.23	88.99%	35.86	38,622	37,171	92,645
Pcan9	3	2.28	34.94	92.11%	34.92	38,558		
Pcan10 *	3	2.56	36.12	96.96%	38.9	40,256	38,844	90,990
Pcan11	3	2.00	35.18	93.17%	31.2	38,551		
Pcan12	3	2.64	35.78	92.91%	40.05	38,563		
Pcan13 *	3	2.56	35.88	95.62%	35.92	38,977	37,539	90,490
Pcan14	3	2.85	35.97	93.41%	42.87	38,540		
Pcan15	3	3.17	36.07	93.63%	43.66	38,550		
Pcan16 *	3	2.83	36.5	97.55%	38.35	39,902	38,405	90,985
Pcan17	3	2.58	37.44	93.35%	37.65	38,543		
Pcan18	3	2.05	35.79	93.25%	30.16	38,546		
Pcan19	3	2.08	35.75	93.52%	30.8	38,546		
Pcan20	3	2.22	35.72	93.61%	32.58	38,547		
Pcan21	3	2.32	35.97	93.69%	34.95	38,551		
Pcan22 *	4	2.86	36.05	96.07%	39.4	34,120	32,941	80,407
Psim1 *	5	2.70	36.33	96.63%	39.52	42,374	40,857	93,843
Psim2	5	2.43	35.01	95.63%	37.35	42,376		
Psim3 *	5	3.01	40.99	97.87%	35.45	44,692	43,147	95,753
Psim4 *	5	2.19	35.87	96.32%	33.46	41,279	39,671	94,038
Psim5 *	5	2.00	35.31	92.77%	31.5	40,952	39,399	94,477
Psim6	5	2.78	36.14	93.61%	39.62	44,683		
Ptom1 *	6	16.18	50.87	48.25%	127.28	1,023,042	1,023,392	
Ptom3 *	6	3.5	37.16	47.38%	30.88	1,023,009	1,023,361	
Ptom2 *	6	3.81	37.06	47.49%	31.46	1,022,870	1,023,223	
Total						1,139,808	1,140,405	430,649

a The asterisk represents nonredundant isolate. b 1, host: *P*. × *euramericana* cv. I214, year: 2015, location: Nanjing; 2, host: *P*. × *canadensis*, year: 2015, location: Nanjing; 3, host: *P*. × *canadensis*, year: 2018, location: Nanjing; 4, host: *P*. × *canadensis*, year: 2018, location: Qiannan; 5, host: *P. simonii*, year: 2017, location: Yan’an; 6, host: *P. tomentosa*, year: 2016, location: Nanjing. c SNP calling with 35 *M*. *brunnea* isolates. d SNP calling with nonredundant 19 *M*. *brunnea* isolates. e SNP calling with nonredundant 16 *MbMu* isolates.

## Data Availability

Clean reads were deposited in the NCBI Sequence Read Archive under BioProject ID PRJNA825296. The assembly of Ptom1 was deposited in GenBank under the accession number GCA_011750725.1. All other assemblies were deposited in CoGe database (https://genomevolution.org/coge/) under the genome ID 63935 (Pcan1), 63936 (Pcan2), 63937 (Pcan3), 63938 (Pcan4), 63939 (Pcan5), 63944 (Pcan6), 63945 (Pcan7), 63946 (Pcan8), 63947 (Pcan9), 63948 (Pcan10), 63949 (Pcan11), 63950 (Pcan12), 63951 (Pcan13), 63952 (Pcan14), 63953 (Pcan15), 63954 (Pcan16), 63955 (Pcan17), 63956 (Pcan18), 63957 (Pcan19), 63958 (Pcan20), 63959 (Pcan21), 63960 (Pcan22), 63961 (Peur1), 63962 (Peur2), 63963 (Peur3), 63964 (Peur4), 63965 (Psim1), 63966 (Psim2), 63967 (Psim3), 63968 (Psim4), 63969 (Psim5), 63970 (Psim6), 63971 (Ptom2) and 63972 (Ptom3). The sequences of *MAT* loci were deposited in GenBank under the accession numbers ON556642 (Peur4), ON556643 (Pcan3), ON556644 (Pcan7), ON556645 (Ptom1) and ON556646 (Ptom5).
